# SLC1A5 glutamine transporter is a target of MYC and mediates reduced mTORC1 signaling and increased fatty acid oxidation in long‐lived *Myc* hypomorphic mice

**DOI:** 10.1111/acel.12947

**Published:** 2019-03-25

**Authors:** Xiaoai Zhao, Anna P. Petrashen, Jennifer A. Sanders, Abigail L. Peterson, John M. Sedivy

**Affiliations:** ^1^ Department of Molecular Biology, Cell Biology and Biochemistry, and Center on the Biology of Aging Brown University Providence Rhode Island; ^2^ Department of Pediatrics Rhode Island Hospital and Brown University Providence Rhode Island; ^3^ Present address: Department of Genetics Stanford University Stanford California

**Keywords:** fatty acid oxidation, genetic models, metabolic regulation, mouse longevity, mTOR signaling, MYC gene

## Abstract

Mice that express reduced levels of the c‐*Myc* gene (*Myc* ^+/−^ heterozygotes) are long‐lived. *Myc* hypomorphic mice display reduced rates of protein translation and decreased activity of the mammalian target of rapamycin (mTOR) complex 1 (mTORC1). Given the prominent effect of mTOR on aging, lower mTORC1 activity could contribute to the exceptional longevity and enhanced healthspan of *Myc* ^+/−^ animals. However, given the downstream position of MYC in these signaling cascades, the mechanism through which mTORC1 activity is downregulated in *Myc* ^+/−^ mice is not understood. We report that the high‐affinity glutamine transporter SLC1A5, which is critical for activation of mTORC1 activity by amino acids, is a transcriptional target of MYC. *Myc* ^+/−^ cells display decreased *Slc1a5* gene expression that leads to lower glutamine uptake and consequently reduced mTORC1 activity. Decreased mTORC1 activity in turn mediates an elevation of fatty acid oxidation (FAO) by indirectly upregulating the expression of carnitine palmitoyltransferase 1a (*Cpt1a*) that mediates the rate‐limiting step of β‐oxidation. Increased FAO has been noted in a number of long‐lived mouse models. Taken together, our results show that transcriptional feedback loops regulated by MYC modulate upstream signaling pathways such as mTOR and impact FAO on an organismal level.

**Abbreviations**
FAO: fatty acid oxidation
GPNA: l‐γ‐glutamyl‐p‐nitroanilide
MHC: primary mouse hepatocytes
MTF: primary mouse tail fibroblasts

The MYC transcription factor is a downstream effector of proliferation and nutrient signaling mediated by receptor tyrosine kinase, RAS, and phosphoinositide 3‐kinase (PI3K) pathways (Dang, 2013). mTORC1 thus occupies a position below PI3K/AKT and above MYC in this signaling hierarchy, yet *Myc* hypomorphic mice display reduced mTORC1 activity in several tissues (Hofmann et al., 2015).

Our transcriptome data suggested that the downregulation of mTORC1 activity in *Myc^+/^* ^−^ mice is unlikely to be due to effects on *Mtor* or *Rptor* gene expression (Hofmann et al., 2015), which was confirmed by RT–qPCR analysis (Supporting Information Figure S1). However, a genome‐wide analysis of the MYC regulome in a rat fibroblast cell line (Yap, Peterson, Castellani, Sedivy, & Neretti, 2011) implicated both the *Slc1a5* and *Slc7a5* genes as direct MYC targets (Supporting Information Figure S2). Given the critical role of glutamine uptake in mTORC1 activation (Nicklin et al., 2009), we asked whether these genes are differentially regulated in *Myc^+/^* ^−^ animals. The expression of *Slc1a5* mRNA in the liver of *Myc^+/^* ^−^ mice was reduced 20%–30% compared to *Myc^+/+^*mice (Figure 1a). This change was observed in vivo as well as in cultured primary mouse tail fibroblasts (MTF) and primary mouse hepatocytes (MHC). A similar downregulation of *Slc7a5* was observed in *Myc^+/^* ^−^ fibroblasts and hepatocytes (Figure 1b).

**Figure 1:**
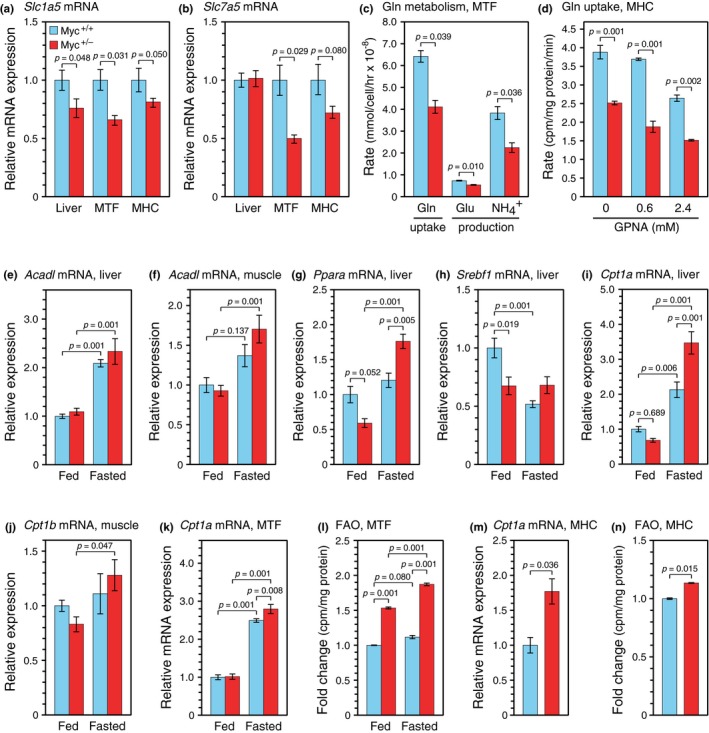
*Myc* haploinsufficiency reduces glutamine uptake and augments FAO in vitro and in vivo. (a) Expression of *Slc1a5* and (b) *Slc7a5* genes. mRNA levels were determined by RT–qPCR and are presented relative to wild‐type (*Myc^+/+^*) liver tissue or primary cells. MTF, mouse tail fibroblasts; MHC, mouse hepatocytes. (c) Glutamine metabolism. The disappearance of glutamine and appearance of glutamate and ammonia in the medium were assessed with the BioProfile FLEX analyzer. (d) Glutamine uptake. Direct uptake of ^3^H‐glutamine was measured by pulsing cultures in glutamine‐free medium for 3 min in the presence or absence of the competitive inhibitor of SLC1A5‐regulated glutamine uptake, GPNA. (e) Gene expression of *Acadl *in liver, (f) *Acadl* in muscle, (g) *Ppara* in liver, (h) *Srebf1* in liver, (i) *Cpt1a* in liver, and (j) *Cpt1b in muscle*. mRNA levels were determined by RT–qPCR under either ad libitum fed or overnight fasting conditions. (k) *Cpt1a* expression and (l) FAO levels in MTF. mRNA levels were determined by RT–qPCR. FAO was quantified using a radioactive assay in which ^3^H‐labeled palmitic acid was provided in the medium, and its oxidation by cells was measured by the release of ^3^H_2_O. For composition of the “fed” and “fasted” media, see Experimental Procedures. (m) *Cpt1a* expression and (n) FAO in MHC. Cell cultures were established from individual animals, 5 months old for MTF, 6–8 weeks for MHC, both sexes, *n* = 3 for all panels. For liver samples, *n* = 5 animals, 10–12 months, females. Error bars represent *SEM*. Statistical significance was assessed using the Wilcoxon rank‐sum test for panels (a)–(c) and (m)–(n), and two‐way ANOVA followed by Tukey's post hoc test for panels (d)–(l)

Reduced *Slc1a5 and Slc7a5* expression was accompanied by a decline in glutamine uptake and metabolism. Relative to *Myc^+/+^*controls, MTF from *Myc^+/^* ^−^ mice showed a 40% decrease in the removal of glutamine from the medium (Figure 1c), as well as a significant decrease in the production of glutamate and ammonia. A quantitative short‐term uptake assay using a radioactive tracer (^3^H‐glutamine) showed a decrease in glutamine transport in *Myc^+/^* ^−^ MHC that paralleled the effects seen in MTF (Figure 1d). We also used l‐γ‐glutamyl‐p‐nitroanilide (GPNA), a competitive inhibitor of SLC1A5‐mediated glutamine uptake (Esslinger, Cybulski, & Rhoderick, 2005), and found that pharmacological inhibition of this transporter resulted in dose‐dependent inhibition of glutamine uptake in MHC. In aggregate, these data show that *Slc1a5* expression is correlated with decreased glutamine uptake in *Myc^+/^* ^−^ cells.

Mammalian target of rapamycin is a nexus for the metabolic regulation of aging.* Myc^+/^* ^−^ mice, which show reduced mTORC1 activity, also have a smaller body size and elevated metabolic activity (increased levels of O_2_ consumption, CO_2_ production, food and water intake, spontaneous activity) when compared to mice with normal level of MYC. Both male and female *Myc^+/^* ^−^ mice exhibit a decrease in the respiratory exchange ratio, particularly under fasting conditions, which suggests that *Myc^+/^* ^−^ mice of both sexes upregulate fatty acid oxidation (FAO) when fasted (Hofmann et al., 2015). To gain more molecular insight of this metabolic change, we examined *Myc^+/+^* and *Myc^+/^* ^−^ mice under either ad libitum feeding conditions, or after overnight (16 hr) fasting prior to sacrifice. Since stored fatty acids are used as a major energy source during fasting, FAO is induced soon after food removal (Arias, Asins, Hegardt, & Serra, 1997; Kersten, Desvergne, & Wahli, 2000). In our experiments, this was evidenced by a twofold upregulation, in both liver and skeletal muscle of fasted animals of both genotypes, of the gene encoding long‐chain‐acyl‐CoA dehydrogenase (*Acadl*), a member of a family of mitochondrial flavoproteins that catalyze the initial steps of β‐oxidation of straight chain fatty acyl‐CoA (Figure 1e,f). We also examined the peroxisome proliferator‐activated receptor alpha (*Ppara*) gene, encoding a transcription factor that regulates ketogenesis (Kersten et al., 2000), and found a fasting‐induced increase in expression similar to that of the *Acadl* gene (Figure 1g).

We then assessed the expression of the sterol regulatory element‐binding protein 1 (*Srebf1*), a key transcriptional regulator that promotes lipogenic genes. Interestingly, the levels of *Srebf1 *were significantly lower in *ad lib *fed *Myc^+/−^* mice compared to *Myc^+/+^* mice, but no differences were observed in fasted mice between the genotypes (Figure 1h). In addition, while *Srebf1 *levels were induced in *ad lib *fed *Myc^+/+^* mice, expression in *Myc^+/−^* mice remained relatively constant under both fed and fasted animals.

To further evaluate changes in FAO, we examined the expression carnitine palmitoyltransferase I genes that control the rate‐limiting steps of long‐chain fatty acid transport into mitochondria (McGarry & Brown, 1997; encoded by distinct *Cpt1a* and *Cpt1b* genes in liver and muscle, respectively). The expression of *Cpt1a* was strongly induced by fasting, especially in liver tissue of *Myc^+/−^* animals (Figure 1i). This was also observed in muscle, albeit with a smaller magnitude of change in *Cpt1b *expression (Figure 1j). Taken together, these data indicate that *Myc^+/−^* mice are more sensitive to starvation than *Myc^+/+^* mice. Notably, genes that are related to lipid catabolism, specifically *Acadl*, *Ppara,* and *Cpt1,* show a larger relative increase in response to fasting in *Myc^+/−^* mice, in contrast to the lipogenic gene *Srebp1*, whose transcriptional level is maintained in *Myc^+/−^*mice regardless of feeding conditions.

To assess whether the augmented upregulation of FAO in the *Myc^+/−^* background could be cell autonomous, we isolated and cultured MTF under two different conditions: in medium containing glucose, pyruvate, and glutamine to simulate a nutrient‐rich fed state and in medium lacking these food sources to simulate a fasted state (both media were supplemented with 10% FBS). *Cpt1a *expression was markedly increased in cells cultured under nutrient‐limiting conditions (Figure 1k). Consistent with fasted tissues, starvation medium elicited a greater increase in *Cpt1a *expression in *Myc^+/−^* cells compared to *Myc^+/+^* cells. To extend this analysis, we performed direct FAO assays using ^3^H‐labeled palmitic acid and quantified the amount of radioactive water that was released by its metabolism. Using this sensitive assay, we found that levels of FAO were significantly higher in cells from *Myc^+/−^* mice grown in both types of media (Figure 1l).

To strengthen the hypothesis of cell‐autonomous upregulation of FAO in *Myc^+/−^* cells, we prepared MHC and found that both *Cpt1a *mRNA levels and FAO were significantly elevated in cells from *Myc^+/−^* mice (Figure 1m,n; we were unable to culture MHC under starvation conditions). In aggregate, these data suggest that the differences in FAO elicited by *Myc *haploinsufficiency have a considerable cell‐autonomous component, since two distinct primary cell types displayed consistent effects in culture. Moreover, unlike the effects in vivo which were only clearly apparent following starvation, FAO levels were significantly elevated in *Myc^+/−^* cells cultured under both fed and fasted conditions.

Given that we have observed a marked decrease in glutamine uptake in *Myc^+/−^* cells, mediated by the direct MYC targets *Slc1a5* and *Slc7a5*, we asked whether these effects on glutamine uptake could be responsible for the attenuated mTORC1 activity found in *Myc^+/−^*cells and tissues. To test this hypothesis, we assessed the activity of S6 protein kinase, a downstream readout of mTORC1 activity, in *Myc^+/+^*MHC treated with GPNA as well as *Myc^+/+^*and *Myc^+/−^*cells treated with vehicle control. Treatment with GPNA decreased levels of S6K activity in *Myc^+/+^*cells and phenocopied the effect of reduced *Slc1a5* expression in *Myc^+/−^* cells (Figure 2a). Hence, mTORC1 activity can be effectively reduced by decreasing SLC1A5‐mediated glutamine uptake.

**Figure 2:**
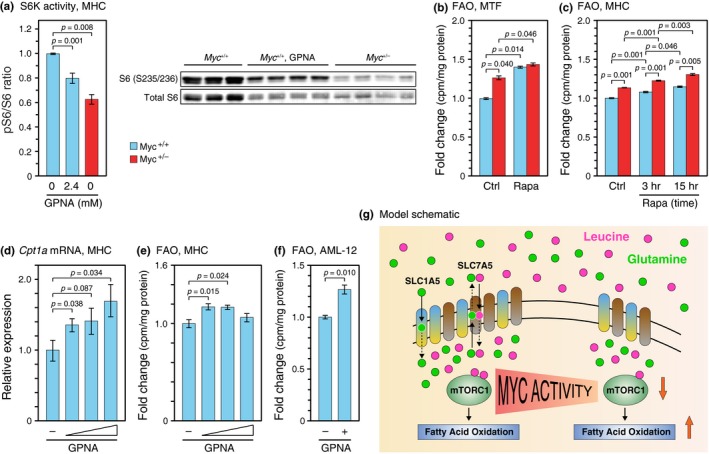
*Myc* haploinsufficiency and pharmacological inhibition of mTOR or SLC1A5 induces FAO. (a) S6K activity. Phosphorylated (Ser235/236) and total S6 ribosomal protein was assessed (left). The ratio between phosphorylated (Ser235/236) to total S6 in the presence or absence of GPNA for 20 hr was quantified (right). (b) Rapamycin treatment increases FAO in MTF and (c) MHC. Cells were cultured in the presence of 100 nM rapamycin (MTF, 15 hr; MHC, as indicated). Cell cultures were established from individual animals, 5 months old for MTF, 6–8 weeks for MHC, both sexes, *n* = 3. (d) GPNA treatment upregulates *Cpt1a* and (e) increases FAO in MHC. MHC were cultured in the absence or presence of 0.3 mM, 0.6 mM, and 1.2 mM GPNA for 24 hr. Cell cultures were established from individual animals as above, *n* = 5 for each treatment group. (f) AML‐12 cells were cultured in the absence or presence of 2 mM GPNA for 24 hr. *n* = 3 independent cultures. Error bars represent *SEM*. Statistical significance was assessed using one‐way ANOVA followed by Tukey's post hoc test for panels (a) and (d), (e), two‐way ANOVA followed by Tukey's post hoc test for panels (b), (c), and Wilcoxon rank‐sum test for panel (f). (g) MYC regulates the mTORC1 pathway through its direct transcriptional targets SLC1A5 and SLC7A5. mTORC1 activity is regulated post‐translationally in part by the cytoplasmic levels of essential amino acids such as leucine. SLC1A5, a high‐affinity glutamine transporter, transports glutamine into cells, some of which is subsequently exchanged for leucine by the SLC7A5 antiporter. *Myc* ^+/−^ cells express the *Slc1a5* and *Slc7a5 *genes at a lower level, which leads to less glutamine uptake, lower intracellular levels of essential amino acids, and consequently reduced mTORC1 activity. Decreased mTORC1 activity in turn mediates an elevation of FAO

mTORC1 activity is decreased in tissues of *Myc^+/−^* mice as well as in cells in culture (Figure 2a), suggesting that at least some of the effects are cell autonomous. To explore whether reduced mTORC1 activity could lead to increased FAO, we treated MTF with the mTORC1 inhibitor rapamycin. As expected, ribosomal protein S6 phosphorylation was decreased by rapamycin treatment (Supporting Information Figure S3). Interestingly, FAO was increased significantly in cells of both genotypes (Figure 2b), and a similar phenomenon was also observed in MHC (Figure 2c). An intermediate time point (3 hr of rapamycin treatment) showed an intermediate effect on FAO. Hence, it appears that inhibition of mTOR increases FAO in a cell‐autonomous and dose‐dependent manner.

Since we were able to inhibit mTORC1 activation by pharmacologically interfering with SLC1A5‐mediated glutamine uptake (Figure 2a), and decreased mTOR signaling in rapamycin‐treated cells induced FAO (Figure 2b,c), we asked whether inhibiting glutamine uptake was sufficient to induce FAO. In these experiments, we used *Myc^+/+^* MHC as well as several cell lines and inhibited SLC1A5 activity with GPNA to phenocopy the effect of reduced MYC levels (*Myc^+/−^* genotype) on *Slc1a5* expression. We found that GPNA increased *Cpt1a *mRNA levels in MHC (Figure 2d), and quantitative assays using ^3^H‐labeled palmitate showed that FAO was concomitantly elevated in the same cultures (Figure 2e). Complementary results were obtained using the TGR‐1 rat fibroblast cell line (Supporting Information Figure S4). Finally, we were also able to increase FAO with GPNA treatment in a third cell culture model, an immortalized mouse hepatocyte cell line (AML‐12, Figure 2f). These data indicate that reduced glutamine uptake is sufficient to downregulate mTOR signaling, which in turn promotes FAO.

In summary, we demonstrate here the existence of an indirect feedback loop by which MYC regulates the transcription of the *Slc1A5* gene, whose gene product in turn modulates the activation status of mTORC1 kinase (Figure 2g). The SLC1A5 transporter regulates the rate‐limiting step of glutamine uptake, with the subsequent exchange of intracellular glutamine for essential amino acids being mediated by SLC7A5 and SLC3A2 (Nicklin et al., 2009). Elevated MYC activity is well known to promote glutamine metabolism in the context of cancer by transcriptionally upregulating several glutamine transporters (including *Slc1A5*) and glutaminase (*Gls*) (Gao et al., 2009; Wise et al., 2008). MYC mRNA and protein levels are both decreased by 40%–60% in Myc^+/^ *^−^* mice and cells (Hofmann et al., 2015); correspondingly, we found *Slc1a5* and *Slc7a5* mRNA to be decreased by 20%–30% and glutamine uptake by 40% in both MTF and MHC. In agreement with our findings that this feedback loop can negatively affect mTORC1 activity, deletion of *Myc *was reported to impair mTORC1 signaling during T‐cell activation (Wang et al., 2011). MYC thus regulates translation by at least two distinct mechanisms: as a direct transcriptional activator of the entire ribosomal biogenesis regulon (Hofmann et al., 2015) and by the indirect feedback loop documented here that affects mTORC1 activation status.

Fatty acid oxidation is significantly elevated in *Myc^+/−^* mice (Hofmann et al., 2015) as well as in other lifespan‐extending interventions, such as dietary restriction (Bruss, Khambatta, Ruby, Aggarwal, & Hellerstein, 2010) or knockout of the growth hormone receptor (Westbrook, Bonkowski, Strader, & Bartke, 2009). FAO was increased by downregulating MYC in a human Burkitt's lymphoma B cell line (Le et al., 2012), as well as by a *Myc *knockout in a rat fibroblast cell line (Edmunds et al., 2014). The large magnitude of these effects was probably due to the large changes in MYC expression explored in these studies: very high starting levels of MYC in Burkitt's lymphoma cells or a complete knockout of MYC in the rat fibroblast cells. In our studies, the more modest 20%–40% increase in FAO probably reflects the 40%–60% reduction of MYC expression in *Myc^+/−^* cells. The cell‐autonomous nature of this effect in both primary MTF and MHC is in agreement with the previous studies.

While fasting‐induced expression of genes regulating lipid catabolism (*Acadl*, *Ppara*, *Cpt1*) was upregulated to a greater extent in liver and muscle of *Myc^+/−^* compared to *Myc^+/+^* mice (Figure 1e–g and i,j), it is interesting to note that expression of the lipogenic transcription factor *Srebf1 *remained largely unchanged regardless of feeding conditions in *Myc^+/−^* mice (Figure 1h). Previous studies have suggested that mTORC1 regulates SREBPs at multiple levels, including transcription, processing, and nuclear accumulation of the active forms of these transcription factors (Bakan & Laplante, 2012). It is thus possible that the post‐transcriptional regulation of SREBF1 by mTORC1 is more pronounced in *Myc^+/−^* mice. In addition, since we observed a marked reduction in AKT and mTORC1 signaling in *Myc* hypomorphic mice (Hofmann et al., 2015), and both pathways promote lipid synthesis (Bakan & Laplante, 2012), it is plausible that *Myc^+/−^* mice maintain a reduced level of lipid synthesis under *ad lib* conditions and do not further decrease it when subjected to short‐term starvation. Further study is required to tease out the possibilities behind this phenomenon.

*Cpt1a *mRNA and FAO levels increased dose‐dependently when MHC were treated with GPNA, a competitive inhibitor of SLC1A5‐regulated glutamine uptake (Figure 2d). The discrepancy between *Cpt1a* mRNA levels and FAO at the highest does of GPNA might be due to toxicity of the drug on primary cells at such high doses. Indeed, when repeating this experiment in both an immortalized mouse hepatocyte cell line AML‐12 (Figure 2f) and the TGR‐1 rat fibroblast cell line (Supporting Information Figure S4), we observed an increase in *Cpt1a* expression and a concomitant elevation in FAO beyond the highest doses used with MHC.

Mammalian target of rapamycin has emerged as an important regulator of lipid homeostasis (Kennedy & Lamming, 2016; Lamming & Sabatini, 2013). Our ability to recapitulate the effect of reduced MYC activity on FAO by treatment of primary cells with rapamycin, or through inhibition of SLC1A5‐mediated glutamine uptake, implicates mTORC1 as an important link between MYC and FAO. In agreement, S6K1 deletion in mice, which impairs one mTORC1‐activated downstream pathway, enhanced FAO and protected animals from obesity (Um et al., 2004). How mTORC1 regulates FAO is not fully understood. mTORC1, or its downstream effector S6K2, can positively affect PPARα activity by promoting the nuclear localization of nuclear receptor corepressor 1 (NCoR1) (Kim, Pyo, & Um, 2012; Sengupta, Peterson, Laplante, Oh, & Sabatini, 2010), but more work is needed to understand the mechanistic details. It would also be of interest to explore additional MYC‐regulated feedback loops that may affect signaling pathways such as AMPK or IGF1, both of which display longevity‐promoting changes in long‐lived *Myc^+/−^* mice.

## CONFLICT OF INTEREST

None declared.

## AUTHOR CONTRIBUTIONS

J.M.S. and X.Z. conceived the study. J.M.S., X.Z., and A.P.P. designed the experiments. X.Z., A.P.P., and A.L.P. performed experiments and analysis. J.A.S. and J.M.S. contributed to data interpretation and critical analysis. J.M.S. and X.Z. wrote the manuscript with feedback from all authors.
