# Supplementary material for: SLC1A5 glutamine transporter is a target of MYC and mediates reduced mTORC1 signaling and increased fatty acid oxidation in long‐lived Myc hypomorphic mice
Source: Aging Cell. 2019 Mar 25;18(3):e12947. doi: 10.1111/acel.12947 (PMC6516164; doi:10.1111/acel.12947)
Supplement: Supplementary file 1 [file ACEL-18-e12947-s001.pdf]

SUPPORTING INFORMATION

Experimental Procedures

Use and Treatment of Animals

Myc$^{+/+}$ and *Myc*$^{+/-}$ mice were produced and housed in a specific pathogen-free AAALAC-certified barrier facility as described (Hofmann et al., 2015). All procedures were approved by the Brown University IACUC committee. Animals of both genotypes and the same sex were housed together.

Harvesting of Tissues

Mice were euthanized in the morning (between 8 and 10 AM) by isofluorane anesthesia followed by cervical dislocation. Animals were brought to the laboratory in their holding cages and euthanized one by one for dissection. The dissection was performed as rapidly as possible following euthanasia by several trained staff members working in concert on one mouse. Major organs were removed, cut into appropriate size pieces, and flash-frozen in liquid nitrogen.

Fragments of tissues in the range of 20-50 mg were removed from samples kept at -80°C, carefully weighed on dry ice, and homogenized in Trizol reagent (Invitrogen) according to the supplier's instructions. Tissue fragments were homogenized directly in Trizol reagent in a Fisher PowerGen 125 motorized homogenizer at room temperature. For RT-qPCR, total RNA was recovered from the Trizol reagent by isopropanol precipitation and further purified using the RNeasy Mini kit (Qiagen). RNA quality was assessed using an Agilent 2100 Bioanalyzer instrument, and the yield was quantified using a NanoDrop 2000 spectrophotometer. 1 μg of total RNA was reverse transcribed into cDNA in 50 μl reactions using the TaqMan kit (Applied Biosystems), according to the manufacturer's protocol. 1 μl of this reaction was used in

subsequent qPCR reactions, which were performed using the SYBR Green system (Applied Biosystems) on the ABI 7900 Fast Sequence Detection instrument, according to the manufacturer's specifications. Primer sequences are listed in Table S1. *Gapdh* (Primer 1), *Hprt* (Primer 9), Beta-actin (*Actb)* (Primer 2), and Beta-2 microglobulin (*B2m*) (Primer 5) were used as internal normalization controls.

Cell Lines and Cell Culture Conditions

AML-12 cells were cultured under normoxic conditions (air supplemented with 5% CO_2; 5% CO_2, 20% O_2, 75% N_2), in a 1:1 mixture of Dulbecco's modified Eagle's medium (DMEM) (Hyclone, SH30243.01) and Ham's Nutrient Mixture F12 (Hyclone, SH30026.01), supplemented with 10% FBS (Hyclone, SH30071.03), ITS supplement containing 0.005 mg/ml insulin, 0.005 mg/ml transferrin, 5 ng/ml selenium (Corning, 354350), and 40 ng/ml dexamethasone (MP Biomedicals, 0219456125). The TGR-1 (*Myc*$^{+/+}$*)* rat fibroblast cell line was cultured in DMEM (Hyclone, SH30243.01) and 10% calf serum under normoxic conditions at 37°C as previously described (Shichiri, Hanson, & Sedivy, 1993). Primary mouse tail fibroblast (MTF) cultures were initiated from 8 to 12-week old *Myc*$^{+/+}$ and *Myc*$^{+/-}$ mice using previously described methods (Harper, Salmon, Leiser, Galecki, & Miller, 2007). MTF were cultured under low oxygen conditions (an atmosphere of 3% O_2, 5% CO_2, and 92% N_2) in DMEM (Hyclone, SH30243.01) supplemented with 10% FBS (Hyclone, SH30071.03). MTF were routinely subcultured at 1:4 dilution upon reaching 80% confluence. Under our conditions MTF reached senescence at passages 11-13; all experiments described here were performed using MTF at passages 1-4.

For the assessment of fatty acid oxidation in MTF, DMEM (Hyclone, SH30243.01), supplemented with 10% FBS was used as "fed media", whereas DMEM without glucose, glutamine or pyruvate (Sigma, D5030), which was supplemented with 10% FBS and 3.7 g/L

sodium bicarbonate, was used as the "fasted media".

Hepatocyte Culture

Establishment of primary mouse hepatocyte (MHC) cultures followed previously published protocols with some modifications (Glick et al., 2012; Klaunig et al., 1981). Specifically, retrograde perfusion was performed by cannulation of the inferior vena cava (IVC). Perfusion buffer (CMF-HBSS, Hyclone, SH3058801, with 0.4 g/L KCl, 1 g/L glucose, 1.8 g/L $NaHCO_3$ and 0.2 g/L EDTA) was introduced at a flow rate of 2-3 ml/min for 2-3 minutes. This was followed by the introduction of the digestion medium (CMF-HBSS, Hyclone, SH3058801, with 5 mM $CaCl_2$, 5 mM HEPES and 1 vial of Collagenase/Elastase mix, Worthington Biochemicals, LK002067, for each 100 ml of digestion buffer). Digestion was stopped when the liver showed signs of disintegration. Liver cells were then dissociated by mincing with forceps and razorblades. Subsequently, hepatocytes were washed in suspension buffer (CMF-HBSS, Hyclone, SH3058801 with 5 mM HEPES) three times at 4°C. Cells were then seeded in DMEM (Hyclone, SH30243.01) supplemented with 10% FBS for 6 hours before switching to serum-free DMEM for the rest of the culture. Culture was under normoxic conditions (air supplemented with 5% CO_2).

Determination of Glutamine, Glutamate and Ammonia Levels in Culture Medium

Appearance of glutamate and ammonia and disappearance of glutamine from culture media were measured using the BioProfile FLEX analyzer (Nova Biomedical) and normalized to cell proliferation rate. Samples for analysis were obtained by adding fresh media to subconfluent cells in 6-well plates and harvesting the media after 24-48 hours of incubation.

Quantitative Fatty Acid Oxidation Assay

The protocol was adapted from Laurent et al. (2013) and Moon and Rhead (1987). MTF, MHC or AML-12 cells were incubated overnight in culture medium containing 100 μM palmitic acid (Sigma, P0500) and 1 mM carnitine (Sigma, C0158). In the final 2 hours of incubation, cells were pulsed with 1.7 μCi of [9,10^{3}H(N)] palmitic acid (Perkin Elmer, NET043001MC) per 1 ml of culture medium. Medium was then collected at the end of the incubation and passed through ion exchange columns packed with DOWEX 1X2-400 resin (Sigma-Aldrich, 217395) to determine the ^3H_2O released by cellular oxidation of [^{3}H] palmitate. Unmetabolized palmitic acid is retained on the column, whereas the ^3H_2O released by its metabolism passes through. The radioactivity in each sample was determined in triplicate using a Beckman LS 5000 TD scintillation counter and liquid scintillation cocktail (Perkin Elmer, Ultima Gold). Fatty acid oxidation was then normalized to the amount of total protein in each sample, which was quantified using the Bradford protein assay (Bio-Rad, 500-0201).

Quantitative Glutamine Uptake Assay

The protocol was adapted from Hassanein et al. (2013). Hepatocytes were incubated in glutamine-free medium with or without L-γ-glutamyl-p-nitroanilide (GPNA) overnight. Cells were then rinsed twice with warm Na^+-free Krebs Ringer Phosphate buffer, in which choline chloride and choline phosphate iso-osmotically replaced the corresponding Na^+ salts (cholKRP) in order to remove extracellular Na^+ and amino acids. Cells were then pulsed with 5 μCi of L-[3,4-^{3}H(N)]-glutamine (Perkin Elmer, NET551001MC) per ml of regular Krebs Ringer Phosphate buffer containing Na^+ (NaKRP), together with 1 mM unlabeled glutamine and incubated at 37°C for 3 minutes. The experiment was terminated immediately after the incubation by rapidly aspirating the incubation solution, followed by washing the cells 3 times with ice-cold PBS. Cells were then lysed with 0.2 M NaOH and 0.2% SDS for 1 hour at room

temperature. Radioactivity in each sample was counted in triplicate using a Beckman LS 5000 TD scintillation counter and liquid scintillation cocktail (Perkin Elmer, Ultima Gold). Glutamine uptake was then normalized to the amount of total protein in each sample, which was quantified using the Bradford protein assay (Bio-Rad, 500-0201).

Statistical analyses

One-way analysis of variance (ANOVA) followed by Tukey's post hoc test was used to assess statistical significance for single-factor comparisons between groups (Fig. 2a, 2d-2e). Two-way ANOVA followed by Tukey's post hoc test was used for multiple-factor comparisons between groups (Fig. 1d-1l, 2b-2c). Two-tailed Wilcoxon rank-sum test was used for single variable comparisons between two groups (Fig. 1a-1c, 1m-1n, 2f and Supplemental Fig. S1 and S4). All statistical analyses were done in R version 3.3.3.

REFERENCES

Glick, D., Zhang, W., Beaton, M., Marsboom, G., Gruber, M., Simon, M. C., . . . Macleod, K. F. (2012). BNip3 regulates mitochondrial function and lipid metabolism in the liver. *Mol Cell Biol, 32*(13), 2570-2584. doi:10.1128/MCB.00167-12

Harper, J. M., Salmon, A. B., Leiser, S. F., Galecki, A. T., & Miller, R. A. (2007). Skin-derived fibroblasts from long-lived species are resistant to some, but not all, lethal stresses and to the mitochondrial inhibitor rotenone. *Aging Cell, 6*(1), 1-13. doi:10.1111/j.1474-9726.2006.00255.x

Hassanein, M., Hoeksema, M. D., Shiota, M., Qian, J., Harris, B. K., Chen, H., . . . Massion, P. P. (2013). SLC1A5 mediates glutamine transport required for lung cancer cell growth and survival. *Clin Cancer Res, 19*(3), 560-570. doi:10.1158/1078-0432.CCR-12-2334

Hofmann, J. W., Zhao, X., De Cecco, M., Peterson, A. L., Pagliaroli, L., Manivannan, J., . . . Sedivy, J. M. (2015). Reduced expression of MYC increases longevity and enhances healthspan. *Cell, 160*(3), 477-488. doi:10.1016/j.cell.2014.12.016

Klaunig, J. E., Goldblatt, P. J., Hinton, D. E., Lipsky, M. M., Chacko, J., & Trump, B. F. (1981). Mouse liver cell culture. I. Hepatocyte isolation. *In Vitro, 17*(10), 913-925.

Laurent, G., German, N. J., Saha, A. K., de Boer, V. C., Davies, M., Koves, T. R., . . . Haigis, M. C. (2013). SIRT4 coordinates the balance between lipid synthesis and catabolism by repressing malonyl CoA decarboxylase. *Mol Cell, 50*(5), 686-698. doi:10.1016/j.molcel.2013.05.012

Moon, A., & Rhead, W. J. (1987). Complementation analysis of fatty acid oxidation disorders. *J Clin Invest, 79*(1), 59-64. doi:10.1172/JCI112808

Shichiri, M., Hanson, K. D., & Sedivy, J. M. (1993). Effects of c-myc expression on proliferation, quiescence, and the G0 to G1 transition in nontransformed cells. *Cell Growth Differ, 4*(2), 93-104.

Yap, C. S., Peterson, A. L., Castellani, G., Sedivy, J. M., & Neretti, N. (2011). Kinetic profiling of the c-Myc transcriptome and bioinformatic analysis of repressed gene promoters. *Cell Cycle, 10*(13), 2184-2196. doi:10.4161/cc.10.13.16249

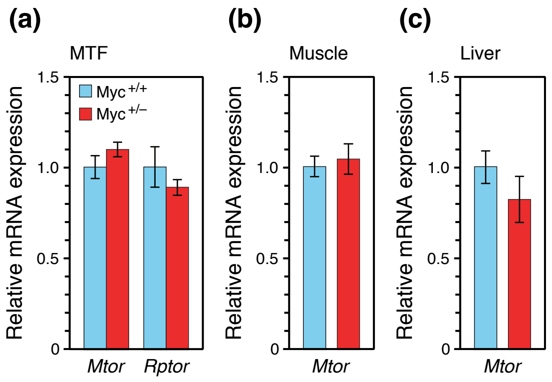

(a)
MTF
Myc +/+
Myc +/−
Relative mRNA expression
1.5
1.0
0.5
0
Mtor
Rptor
(b)
Muscle
Relative mRNA expression
1.5
1.0
0.5
0
Mtor
(c)
Liver
Relative mRNA expression
1.5
1.0
0.5
0
Mtor

FIGURE S1. Expression of *Mtor* and *Rptor* genes in cells and tissues. (a) *Mtor and Rptor* expression in MTF. (b) *Mtor* expression in muscle. (c) *Mtor* expression in liver. mRNA levels were determined by RT-qPCR. Cell cultures were established from individual animals, 5 months old for MTF, both sexes, n=3. For muscle and liver samples n=5 animals, 10-12 months, females. Error bars represent SEM. Statistical significance was assessed using the Wilcoxon rank-sum test. None of the comparisons were significant.

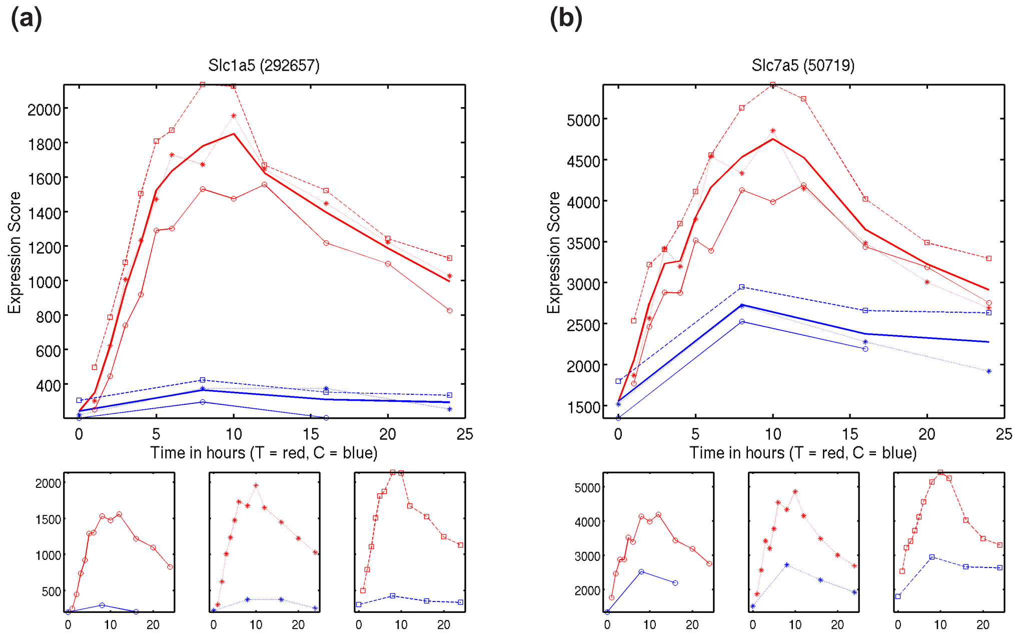
(a)
(b)
Slc1a5 (292657)
Slc7a5 (50719)
Expression Score
Time in hours (T = red, C = blue)

FIGURE S2. *Myc*-induced gene expression changes. mRNA expression changes of (a) *Slc1a5* and (b) *Slc7a5* after induction of *Myc* for 0-24 hours in HOMycER12 cell line (Yap, Peterson, Castellani, Sedivy, & Neretti, 2011). This cell line has been deleted for both endogenous copies of the *Myc* gene and further engineered to express a tamoxifen-inducible *MycER* transgene. Hence, MYC activity can be rapidly regulated from essentially zero to supra-physiological levels by addition of tamoxifen to the medium. Expression patterns of cells from all replicates with (red) or without (blue) *Myc* induction is shown at the top. Each individual biological replicate is shown in three separate lower panels. Note the ~7- and ~3-fold increases in the expression of the *Slc1a5* and *Slc7a5* genes, respectively. The rapid kinetics of induction are indicative of direct regulation by MYC. For full dataset on all genes see: www.brown.edu/Project/Myc.

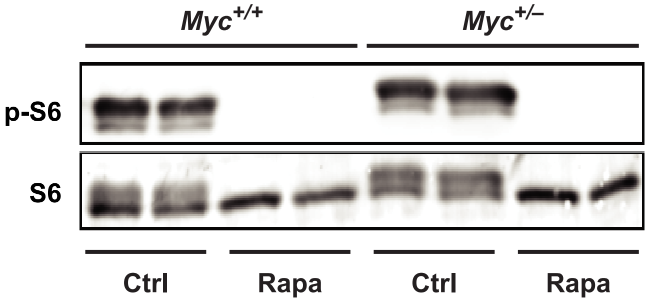

Myc+/+
Myc+/–
p-S6
S6
Ctrl
Rapa
Ctrl
Rapa

FIGURE S3. Pharmacological inhibition of mTOR signaling in MTFs. Western blotting of phosphorylated-S6 (Ser235/236) and total S6 ribosomal protein in MTFs with or without 100nM rapamycin treatment for 15 hours. *n*=3 independent cultures.

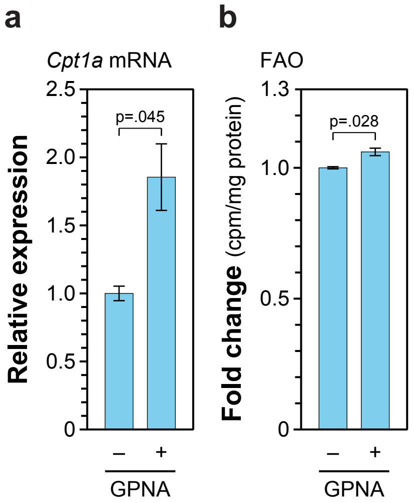
a
Cpt1a mRNA
Relative expression
2.5
2.0
1.5
1.0
0.5
0
p=.045
–
+
GPNA
b
FAO
Fold change (cpm/mg protein)
1.3
1.0
0.5
0
p=.028
–
+
GPNA

FIGURE S4. Pharmacological inhibition of SLC1A5 promotes fatty acid oxidation in the TGR-1 rat fibroblast cell line. (a) GPNA treatment upregulates *Cpt1a* and (b) increases FAO in TGR-1 cells. mRNA levels were determined by RT-qPCR and FAO was determined using the ^{3}H-palmitic acid assay. TGR-1 cells were cultured in the absence or presence of 2 mM GPNA for 20 hours. n=3 independent cultures. In all panels error bars represent s.e.m. Statistical significance was assessed using Wilcoxon rank-sum test.

TABLE S1

List of Primers Used in PCR Analysis[1]

Primer pair 1	MGAPDHF<br>MGAPDHR	CGGCCGCATCTTCTTGTG<br>GTGACCAGGCGCCCAATA	Sense orientation.<br>Antisense orientation.<br>Used for RT-qPCR of the mRNA of the *Gapdh* gene. Primers span an intron.
Primer pair 2	MACTBF<br>MACTBR	GCAAGCAGGAGTACGATGAGT<br>AGAAAGGGTGTAAAACGCAGC	Sense orientation.<br>Antisense orientation.<br>Used for RT-qPCR of the mRNA of the *ActB* gene. Primers span an intron.
Primer pair 3	XZMLCADBF<br>XZMLCADBR	CGATCGCCTGCCCATGGCATT<br>GCTGTGTCCTGAGCTTTCATTCCC	Sense orientation.<br>Antisense orientation.<br>Used for RT-qPCR of the mRNA of the *Lcad* gene. Primers span an intron.
Primer pair 4	XZMSREBF1BF<br>XZMSREBF1BR	ACTTTTCCTTAACGTGGGCCT<br>CATCTCGGCCAGTGTCTGTT	Sense orientation.<br>Antisense orientation.<br>Used for RT-qPCR of the mRNA of the *Srebf1* gene. Primers span an intron.
Primer pair 5	XZMB2M2F<br>XZMB2M2R	CTACTCGGCGCTTCAGTCG<br>ATACAGGCCGGTCAGTGAGA	Sense orientation.<br>Antisense orientation.<br>Used for RT-qPCR of the mRNA of the *B2m* gene. Primers span an intron.
Primer pair 6	XZMCPT1A1F<br>XZMCPT1A1R	ACACCACTGGCCGCATGTCA<br>GCACCTTCAGCGAGTAGCGCA	Sense orientation.<br>Antisense orientation.<br>Used for RT-qPCR of the mRNA of the *Cpt1a* gene. Primers span an intron.
Primer pair 7	XZMCPT1B2F<br>XZMCPT1B2R	TTTGGTCCCGTGGCGGATGA<br>CCAAAGCGCTGGGCGTTCGT	Sense orientation.<br>Antisense orientation.<br>Used for RT-qPCR of the mRNA of the *Cpt1b* gene. Primers span an intron.
Primer pair 8	XZMPPARA2F<br>XZMPPARA2R	CACGCGTGCGAGTTTTCAGG<br>CCCCCAAAACAGCTGCGAAC	Sense orientation.<br>Antisense orientation.<br>Used for RT-qPCR of the mRNA of the *Ppara* gene. Primers span an intron.
Primer pair 9	XZMHPRT1F<br>XZMHPRT1R	CTGTGGCCATCTGCCTAGTA<br>GGGACGCAGCAACTGACATT	Sense orientation.<br>Antisense orientation.<br>Used for RT-qPCR of the mRNA of the *Hprt* gene. Primers span an intron.
Primer pair 10	XZMSLC1A51F<br>XZMSLC1A51R	CACTGCTTTCGGGACCTCTT<br>CCGATGGGTAGGATGAACCG	Sense orientation.<br>Antisense orientation.<br>Used for RT-qPCR of the mRNA of the *Slc1a5* gene. Primers span an intron.
Primer pair 11	XZMSLC7A52F<br>XZMSLC7A52R	ATGACGCTGATGTACGCCTT<br>CGGAGCCACATCATACCGAT	Sense orientation.<br>Antisense orientation.<br>Used for RT-qPCR of the mRNA of the *Slc7a5* gene. Primers span an intron.

[1] All sequences are listed in the 5'->3' orientation.
